# Could Provider Bias Play a Role in Gynecological Health, Sexual Health and Gynecological Cancer Disparities Observed Among a Cohort of Non-English-Speaking Women with HIV living in Southern Florida?

**DOI:** 10.18103/mra.v12i11.6056

**Published:** 2024-11-29

**Authors:** Lunthita M. Duthely, Rachel Mpanumpanu, Isabel Maldonado, Beverly Goldsmith, Isabelle I.M. Akinyemiju, Yulie Lugo, Elena Cyrus

**Affiliations:** 1 University of Miami School of Medicine, Department of Obstetrics, Gynecology and Reproductive Sciences; 2 Ross University School of Medicine; 3 University of Miami, School of Nursing and Health Studies; 4 University of Miami School of Medicine; 5 Indiana University School of Medicine, Department of Internal Medicine; 6 University of Central Florida, Department of Population Health Sciences

## Abstract

As part of an ongoing, prospective study developing an HIV adherence and engagement intervention for women in Southern Florida, we abstracted baseline demographic, psychosocial and medical history data charted in the participants’ electronic medical records. Several differences were observed, in terms of documentation of gynecological and sexual and health data by patients’ linguistic preference. The purpose of this quantitative, retrospective study was to test the differences of data documentation by linguistic group and comment on the findings.

## Background

The Ending the HIV Epidemic (EHE) initiative in the United States (US), seeks to reduce new HIV infections by 75% by the year 2030 ^[1]^. This multi-agency, national initiative concentrated on metropolitan regions in the US most impacted by HIV by infusing additional federal funding to expand effective prevention and treatment strategies. Heterosexual women living in Florida are at an increased risk for HIV because the US South continues to be one of the most affected EHE regions in the US. Heterosexual women are approximately three times more likely to acquire HIV, compared to heterosexual men ^[2]^. Women with HIV face other co-morbid conditions such as gynecological cancers, so new models of care have been implemented to screen early for cancers that are rare in the general population, but higher among women with HIV, like anal cancer ^[3]^.

There are several reasons why women with HIV may not be actively engaged in care. Barriers to care include fewer financial and health care resources, fear of partner retaliation, abuse/violence, transportation, unstable housing and homelessness, lack of emotional or physical support and caregiving responsibilities for both minors and adult family members ^[4]^. At the intersection of race, HIV, poverty and low-income, racial/ethnic minoritized women with HIV in the southeastern US experience several barriers to HIV care. Common barriers include financial challenges and delayed treatment until the onset of symptoms ^[5]^. Inequities are the starkest among racial/ethnic minoritized women in the Southern regions of the US. Many of these inequities can be linked directly to social determinants of health. Structural issues, unequal access to resources, mistrust towards the American health care system and linguistic barriers, for non-native English-speakers, are cited as the most significant barriers to care ^[6]^. The highest proportion of women dying from HIV in the US are women who self-identify as African-American/Black (AA/B) ^[7]^ and Hispanic/Latina ^[8]^. HIV stigma is another challenge faced by people with HIV in the southeastern US. AA/B women may originate from different ethnic and linguistic backgrounds; however; population data are not always delineated further by ethnicity or language. The State of Florida is one of the most racially/linguistically/ethnically diverse states in the US. Our recruitment site is located in Miami-Dade County, where 75% of residents speak a language other than English at home ^[9]^.

The majority of foreign-born people in Florida are from the Latin American and Caribbean (LAC) region. One in five Floridians are born outside of the US—hailing mostly from the Caribbean, and Central and South America (Cuba 23%, Haiti 8%, Colombia 6%, Mexico 6% and Jamaica 5%) ^[10]^. People of Hispanic/Latino descent represent one of the largest minoritized groups in Florida. According to the 2023 U.S. Census Bureau, Hispanic/Latinos comprise about 30% of Florida’s population, and nearly 70% of Miami-Dade County’s population ^[9]^. The Hispanic/Latino population in Florida is disproportionately affected by HIV. In 2022, the HIV diagnosis rate for Hispanic/Latinos in Florida was 22.4 per 100,000, verses 17.9 per 100,000 for non-Hispanic/Latinos ^[11]^. Among foreign-born Black people, people of Haitian descent are one of the largest groups in Florida (about 2.12% of the state’s population and over 400,000 Haitian Creole speakers) ^[12]^. In Miami-Dade County, Haitians are disproportionately affected by HIV and account for 9% of HIV cases and 16% of AIDS cases ^[12]^.

The US has made great strides in lowering HIV prevalence by improving access to HIV testing, HIV prevention and HIV treatment--thereby improving overall HIV viral suppression rates. Furthermore, compared to a decade ago, HIV medications come with a lower pill burden and are less toxic. As people with HIV are living longer, there is the potential of developing other co-morbid conditions as they age, including gynecological and reproductive comorbidities for women. One challenge faced by women with HIV in the US is an elevated risk of anogenital cancer—cancers of the anus and genital tract. In fact, in the State of Florida, where our clinic is located, women of Black race are in the highest risk categories of women developing anogenital cancer and dying of these conditions, compared to Hispanic/Latina women and women of other races and ethnicities ^[13]^. Provider biases and assumptions have been shown to contribute to these inequities ^[14]^. To demonstrate this further, we direct the reader to research that has shown that thorough and accurate diagnosis and documentation of patient gynecological and sexual health by Ob/Gyn providers is strongly tied to the religious, personal, and cultural views of the provider ^[15]^.

### GYNECOLOGICAL AND REPRODUCTIVE COMORBIDITIES AMONG AGING WOMEN WITH HIV IN THE AMERICAS

Migration from Latin America/Caribbean (LAC) to the US has resulted in the State of Florida becoming one of the most diverse States in the US and rates of HIV are influenced by this migration. Women from the Caribbean have higher rates of HIV acquisition compared to women in North, South, and Central America ^[16 17]^. In the US, HIV is widely studied among men, but understudied among women. However, increases in public health programs and research have resulted in a better understanding of HIV among women—more specifically among racial/ethnic minoritized women, where the epidemic is the highest ^[18]^. As of 2022, 18% of HIV diagnoses occurred among women, and the highest incidence is among African-American/Black (AA/B) (39%) and Hispanic/Latina (H/L) (31%) women, compared to White (24%) women ^[19]^. Additionally, in 2022, 22% of women with HIV died from any cause ^[19]^. More specifically, African American women have consistently been at a disproportionate HIV risk, compared to other US women ^[5]^. According to the CDC, cis-gendered AA/B women accounted for 91% of all new HIV infections in the US and H/L women accounted for 19% with the greatest concentration documented in the Southern US ^[19]^.

### BARRIERS TO HIV AND GYNECOLOGICAL AND SEXUAL HEALTH CARE FOR RACIAL/ETHNIC MINORITIZED WOMEN IN THE US

#### Racism.

Structural racism contributes to inequities in HIV-related health outcomes for racial/ethnic minoritized women in the US. In the US, women of racial/ethnic minoritized status represent the highest proportion of women with- and dying from- HIV ^[7]^. They also face race- and HIV-related discrimination and gendered racial microaggressions. These microaggressions include comments related to both gender and race that contribute to exacerbating the community stigma towards racial/ethnic minoritized women with HIV ^[7]^. Other research conducted among low-income racial/ethnic minoritized women with HIV in the Southeastern US found that these women identified racism as a barrier to HIV care ^[5]^.

#### Mistrust of Providers and Medical Systems.

Mistrust towards the American healthcare system was one of the most significant barriers to care identified by a cohort of AA/B women in Philadelphia ^[6]^. In another study among low-income AA/B women with HIV in the southeastern US, women identified trustworthiness of the healthcare system as a barrier to care ^[5]^. In our prior work, we found medical mistrust to be correlated to lowered adherence to ARV medications, across multiple measures of adherence, among English-, Spanish-, and Haitian Creole-speaking women ^[20]^. Another study reported that among Haitian women with HIV, many initiated HIV care in pregnancy, when they were first diagnosed ^[21]^.

#### Stigmatization of HIV/AIDS in the US.

At the start of the epidemic (1981), misinformation and misconceptions regarding the virus drove widespread stigmatization of the virus and treatment. For example, communities believed that HIV only affected men who have sex with men (MSM). Further, after this myth was dispelled and the scientific community elucidated risks associated with heterosexual transmission, it was then proposed that the HIV virus emerged from the Caribbean country of Haiti into the US ^[22 23]^. Both beliefs contributed, unfortunately, to the stigmatization of HIV and created a negative response from the public that has persisted, even in contemporary times. Consequently, at one point, all MSM and people of Haitian origin, regardless of HIV serostatus, were banned from donating blood. Additionally, due to misinformed policy, there was a travel ban in the US in 1987, restricting people with HIV from travelling the US, which was not overturned until 2010 ^[24]^.

HIV continues to be a serious public health concern in the US and is a high priority for research. The 2022 CDC HIV Surveillance report reported the HIV diagnosis rate is at 13.3 per 100,000 population in the US ^[19]^. Over time, evidence-based approaches to determining HIV risk replaced homophobic and racist practices. Behaviors that increase risk include, for example, unprotected/condomless sex with someone who has HIV and sharing needles with someone who has HIV. Nevertheless, the stigmatization of HIV in the US and worldwide continues. For women with HIV, this stigmatization is a barrier to them seeking healthcare, including sexual and reproductive healthcare which, downstream, results in poorer health outcomes– including both HIV- and non-HIV-related co-morbidities ^[25–27]^.

### ELEVATED CANCER RISK FOR WOMEN WITH HIV

Cancer is a major cause of death for people with HIV. In the US, about 1 in 4 people with HIV, co-diagnosed with cancer, are women ^[28]^. Cancer has imposed major health burdens across the globe among American, Caribbean, and European women, and is internally a leading cause of death ^[29 30]^. Women with HIV are at elevated risk of anogenital cancer ^[31]^. Black non-Hispanic and Hispanic women have the highest incidence rates of cervical cancer ^[32 33]^.

Due to the elevated risk of cervical and other genital cancers among women with HIV, women with HIV are screened regularly for cervical cancer. Although considered to be relatively rare in general population of women, anal cancer prevalence is high among women with HIV. Screening for anal cancer, however, lags behind cervical cancer screening ^[34]^. The State of Florida, located in the Southern US, has one of the highest rates of anogenital cancers in the country. The clinic from which participants were enrolled, embarked on routine anal cancer screening, referral and treatment, approximately 10 years ago ^[3]^.

As part of an ongoing study developing an HIV adherence and engagement intervention for a multi-lingual population of women in Southern Florida, we collected gynecological, sexual and health data from the participants’ medical record. This paper summarizes the findings, highlighting the differences by linguistic group.

## Methods

### Design and sample:

As part of an ongoing, larger study, examining barriers and facilitators to HIV care, retrospective, cross-sectional psychosocial, gynecological and sexual history were extracted from institutional electronic medical records (EMRs) and databases from a cohort of racial/ethnic minoritized women with HIV, receiving care in a South Florida clinic. Participants also completed several questionnaires. Baseline demographic profile (age, ethnicity), behavioral health (alcohol, smoking and illicit drug use), mental health history, gynecological profile and sexual history were collected for cis-gendered women with HIV, (n=54), at risk for anal cancer.

### Procedures:

This study was approved by the Institutional Review Board (IRB) of the primary author’s institution. Women were approached by the research team at the time of their clinical appointment. Women who agreed to participate signed an informed consent form for the larger, ongoing parent study. The data for this study was extracted from their medical records.

## Results:

### Description of the Cohort:

Descriptive statistics were used to summarize the data from the EMR. The mean age of the cohort was 45 years old. 51.9% were English speakers, 22.2% were Creole speakers, and 25.9% were Spanish speakers. The average age of the women was 44.6 years. Tobacco, alcohol and illicit drug use within the past 12 months was reported by 14%, 10% and 14%, of the women, respectively. Nearly 40% had a history of a diagnosed mental health condition.

### Comparisons by Linguistic Group:

The majority were heterosexual (97%); however, data were missing across all three linguistic groups (Table 2). Compared to English speakers, generally clinical records for gynecological and sexual history were incomplete for a larger proportion of non-English speakers (Table 2).

Documentation regarding sexual orientation was missing for 29% of English-speakers, 50% of Spanish-speakers and 67% of Haitian Creole-speakers. EMR review showed that the majority were sexually active (59%), however documentation was missing for 14% of English speakers, 7% of Spanish speakers, and 25% of Haitian Creole-speakers. Regarding life stage status, it was documented that the majority (64%) were pre-menopausal; however, documentation was incomplete for 11% of English-speakers; 14% of Spanish-speakers and 17% of Haitian Creole-speakers. Approximately 35 participants were screened for anal cancer, where 33% of English-speakers had abnormal histology results and 25% of both the Spanish- and Haitian Creole-speakers had abnormal histology results; however, 53% of English-speakers (8 of 15) and 33% of Spanish speakers (4 of 12) and 25% (2 of 8) of Haitian Creole speakers had follow-up biopsy results, from a high resolution anoscopy (HRA) procedure required for abnormal histology findings (Table 3).

### EMR Documentation by Linguistic Group:

We then compared the EMR documentation of gynecological and sexual history, dichotomized as *documented* vs. *not documented*, by linguistic group. Statistical analyses were performed in Excel. Differences were tested using the most appropriate test—chi-square (*X*^2^) test or Fisher’s Exact test. A p-value < 0.05 was set as significance with 95% confidence intervals. For each of the three variables, sexual orientation, sexual activity and menopause status, there was a higher proportion of missingness in the EMR for the Haitian Creole speakers. With the exception of the documentation regarding participants’ sexual activity, Spanish speakers had the next highest amount of missingness (i.e. sexual orientation and menopause status). Differences were significant for sexual activity documentation for English vs Spanish vs Creole speakers (p-value =0.00, Table 4).

## Discussion

In summary, we found missing gynecological and sexual health information that differed by patients’ primary language (English, Spanish or Haitian Creole), and that missing information occurred more often in the electronic medical records (EMRs) of non-English speakers. Furthermore, the missingness was more pronounced for the Haitian Creole-speakers, across all data that were collected. We also found differences in follow-up for women with anogenital (anal canal or genitals) dysplasia. Anogenital dysplasia is a cancerous pre-condition. Regarding anal cancer screening and follow-up, Spanish speakers and Haitian Creole-speakers with dysplasia, had lower rates of high resolution anoscopy (HRA) results, 25% and 33%, respectively—suggesting higher lost-to-follow-up for HRA amongst non-English speakers, compared to English speakers.

Linguistic-related challenges with providers have been noted in other studies we reviewed, for people born outside of the US. Linguistic barriers can lead to miscommunication between the provider and the patient. Language, a defining feature of culture, heavily influences communication in the hospital or clinic setting ^[35]^. A discordance in language between a patient and provider may lead to increased dissatisfaction from the patient ^[36]^. Some patients may even delay seeking care if they do not trust the clinic or hospital staff.

In reviewing the literature, we found other studies had reported bias from Ob/Gyn providers, when evaluating patient sexual health. Ob/Gyn providers’ assessments were strongly tied to their religious, personal, and cultural views, as well as their perceived adequacy and comfort levels. One qualitative study conducted in the US found that Ob/Gyn providers who prioritized religion in their own lives were more likely to disapprove of their patients’ sexual practices. They also encouraged patients to adopt different sexual behaviors ^[15]^. These same providers were also less likely to ask about sexual activity if they believed the patient would reveal something that went against their (providers’) own religious beliefs. One study found that Ob/Gyn providers who reported being comfortable with their own sexuality and more satisfied in their sexual lives were more likely to discuss sexual health topics with their patients ^[37]^. A review from Kingsberg et al. found that providers may not offer adequate sexual health care due to the stigma that surrounds the topic of sex, in general. Healthcare providers may not offer sexual health care to older women due to the societal assumption that “women’s sexual function is less relevant beyond reproductive years” ^[38]^. Providers may also experience discomfort in talking about sexual health with patients from a different ethnicity, culture, or sexual orientation than themselves ^[39]^. One Swedish study found that Ob/Gyn providers assumed their patients were heterosexual, failing to acknowledge differences in needs of patients of different sexual orientations ^[40]^. One important consideration is that providers have attributed their discomfort discussing sexual health to inadequate training in sexual health and well-being. A Finnish study found that 92% of Ob/Gyn providers wanted more education on sexual health and well-being. This was expressed more by providers in the 40–49 age group, compared to providers in the 28–39 age group ^[41]^.

We recognize that our findings are limited by the retrospective nature of the data collection, which relied on medical care documentation and notes recorded in the patients’ EMR. Also, the data reviewed encompassed information documented within one year of when the participants’ baseline data were collected. Despite these limitations, our findings that missing data and incomplete data were more pronounced for non-English speakers are important.

First, poor provider communication regarding gynecological and sexual health can be a contributing factor to poor health outcomes for racial/ethnic minoritized and other marginalized women. One study found that South Asian women, despite being part of high-income groups and being adequately insured, were less likely to receive cervical cancer screening in the form of pap smears (Nguyen et al. 2003). The same study found that these women had increased odds of receiving cervical cancer screening if they received clear instructions ^[42]^.

Another study found that Black/African-American patients skipped follow up appointments to their cervical cancer screening, when medical jargon was used and when the information that was provided lacked simple explanations ^[43]^. Another important aspect is that women are more likely to follow-up and complete their cervical screenings when providers included them in the decision-making process about their health ^[44]^. Second, errors in medical record documentation may further exacerbate health disparities already experienced by racial/ethnic minoritized populations and other marginalized populations ^[45,46]^. Documentation errors, including incorrect data entry or incomplete information, can lead to improper treatment, misdiagnoses, and other adverse health outcomes ^[45]^. A recent study demonstrated that despite efforts made by the Institute of Medicine (IOM) over 20 years to address health disparities, data collection for minority groups remains incomplete, likely due to socially constructed identities. To address these persistent issues, implementation of training programs for EHR documentation quality is essential ^[47]^.

## Conclusion

The US Ending the HIV Epidemic (EHE) initiative seeks a dramatic reduction in HIV transmission by 2030. However, continued work is needed to reduce disparities—both in HIV transmission and in reducing HIV-related co-morbid conditions, especially among heterosexual women with HIV—a growing demographic among persons with HIV. HIV is a stigmatizing condition, rooted in misinformation from the early days of the epidemic. Racial/ethnic minoritized persons with HIV may face compounding biases and stigmas related to their condition and their racial/ethnic/linguistic background. Physicians are at the center of the care and treatment for persons with HIV, as well as the prevention of new infections. Our retrospectively gathered, cross-sectional data revealed disparities by linguistic group, regarding the completeness of gynecological- and sexual heath-related information. Specifically, non-English speakers had higher rates of missing information for menopausal status, sexual orientation and sexual activity—important documentation needed during the medical care visit and for continuity of obstetrical/gynecological care. It is important for Obstetricians/Gynecologists, who bare the bulk of the responsibility of the sexual and reproductive health of women, to be aware of their personal, cultural and religious beliefs, which may bias the care they provide to patients—specifically, patients with HIV. The elimination of HIV will require that medical care teams provide the highest quality care possible to people with- or at-risk for- HIV. Training may be needed to ensure the care is culturally competent and free of bias.

## Figures and Tables

**Table 1.** Participant Demographic and Psychosocial Profile (N=54)

	Frequency (n)	Mean (SD) or Column %
**Age (Mean)**	**~**	44.6 (12.7)
**Language Preference**		
English	28	51.9
Spanish	14	25.9
Haitian Creole	12	22.2
**Highest Level of Education Attainment (n=51)**		
Middle School or Less	13	25.5
High School/GED	33	64.7
Post-Secondary	5	9.8
**Tobacco Use (n=51)**		
Yes (12 months)	7	13.7
**Alcohol Use (n=51)**		
Yes (12 months)	5	9.8
**Drug Use (n=51)**		
Yes (12 months)	7	13.7
**Mental Health History (n=51)**		
Major Depressive/Psychiatric Disorder (Yes)	19	37.3

**Table 2:** Participant Sexual and Gynecological Profile by Linguistic Group (N=54)

	English Speakers n (%) (n=28)	Spanish Speakers n (%) (n=14)	Haitian Creole Speakers n (%) (n=12)
**Sexual Orientation**			
Heterosexual	19 (67.9)	7 (50.0)	4 (33.3)
Homosexual	1 (3.6)	0 (-)	0 (-)
Not Documented	8 (28.6)	7 (50.0)	8 (66.7)
**Sexual Activity**			
Abstinent	5 (17.8)	8 (57.1)	6 (50.0)
Yes, sexually active	19 (67.8)	5 (35.7)	3 (25.0)
Not Documented	4 (14.4)	1 (7.1)	3 (25.0)
**Menopause Status**			
Post-Menopausal	4 (14.2)	8 (57.1)	4 (33.3)
Pre-Menopausal	19 (67.8)	4 (28.5)	5 (41.6)
Hysterectomy	2 (7.1)	0 (-)	1 (8.3)
Not Documented	3 (10.9)	2 (14.4)	2 (16.8)

**Table 3:** Participant Gynecological Anal Cytology and Pathology History by Language

	English n (%)	Spanish n (%)	Haitian Creole n (%)
**Documented Anal Cytology (Pap) Results by Language (N=35)**			
Negative	0	1 (8.3)	0 (-)
ASCUS	10 (66.7)	8 (66.7)	6 (75.0)
LSIL/HSIL	5 (33.3)	3 (25.0)	2 (25.0)
* Total*	15	12	8
**Documented Follow-up Pathology (HRA) Results (N=14)**			
Negative	2 (25.0)	0 (-)	0 (-)
AIN-1	3 (37.5)	2 (50.0)	2 (50.0)
AIN-2+	3 (37.5)	2 (50.0)	2 (50.0)
* Total*	8	4	4

**Table 4.** Sexual Orientation, Sexual Activity, Menopause Status Documentation by Linguistic Group

	English n (%)	Spanish n (%)	Haitian Creole n (%)
**Sexual Orientation**			
Documented	20 (71.4)	7 (50)	4 (33.3)
Not Documented	8 (28.5)	7 (50)	8 (66.6)
*Total (p > 0.05)*	28	14	12
**Sexual Activity**			
Documented	24 (85.7)	13 (92.8)	9 (75.0)
Not Documented	4 (14.3)	1 (7.2)	3 (25.0)
*Total (p = 0.00)*	28	14	12
**Menopause Status**			
Documented	25 (89.3)	12 (85.7)	10 (83.3)
Not Documented	3 (10.7)	2 (14.3)	2 (16.6)
*Total (p > 0.05)*	28	14	12
